# Multisystem Inflammatory Syndrome in a Young Adult Following COVID-19 Infection: A Case Report

**DOI:** 10.7759/cureus.24042

**Published:** 2022-04-11

**Authors:** Brian N Bartlett, Abraham Joseph, Anwar Khedr, Hisham Ahmed Mushtaq, Abbas B Jama, Mohamed Hassan, Nitesh K Jain, Syed Anjum Khan

**Affiliations:** 1 Emergency Medicine, Mayo Clinic Health System, Mankato, USA; 2 Hospital Medicine, Mayo Clinic Health System, Mankato, USA; 3 Critical Care Medicine, Mayo Clinic Health System, Mankato, USA

**Keywords:** cardiogenic shock, meningitis, covid-19, multisystem inflammatory syndrome in children (mis-c), multisystem inflammatory syndrome in adults [mis-a]

## Abstract

Multisystem inflammatory syndrome (MIS) after a primary infection with coronavirus disease 2019 (COVID-19) was first recognized in 2020 and presents with similar symptoms as Kawasaki disease, toxic shock syndrome, and macrophage activation syndrome/secondary hemophagocytic lymphohistiocytosis. In children, it is called multisystem inflammatory syndrome in children (MIS-C); in adults, it is termed multisystem inflammatory syndrome in adults (MIS-A). This case offers a unique presentation of MIS in a 20-year-old young adult, who turned 21 years old one week after his presentation. He fits the criteria for MIS-C and MIS-A according to the Centers for Disease Control and World Health Organization, respectively. Initial symptoms in the emergency department included headache, neck stiffness, and fever with diffuse rash. Other symptoms consistent with MIS-C/A developed rapidly later during the course of the disease.

## Introduction

Coronavirus disease (COVID-19) illness is caused by the severe acute respiratory syndrome coronavirus-2 (SARS-CoV-2). COVID-19 emerged in late 2019 and was designated a pandemic in March 2020, prompting the establishment of worldwide mitigation policies to halt the disease's spread and the initiation of a global effort to unravel the etiology, discover successful cures, and produce safe and effective vaccines [1].

Children and adolescents are just as vulnerable as adults to SARS-CoV-2 infection, although they have a much lower rate of symptomatic COVID-19 primary infection and seldom develop severe illness [2,3]. However, four to six weeks following infection with primary COVID-19, a small percentage of children develop a life-threatening hyperinflammatory condition known as a multisystem inflammatory syndrome in children (MIS-C) [4]. A similar syndrome has been documented as a rare COVID-19 adult complication (MIS-A) [5]. Additionally, vaccine-related MIS (MIS-V) has also been reported in the medical literature [6,7].

Herein, we present a case of a multisystem inflammatory syndrome (MIS) in an unvaccinated young 20-year-old young adult that initially mimicked meningitis and lacked sufficient criteria for a diagnosis of MIS. Throughout the workup, other symptoms of MIS developed rapidly. As a relatively new diagnosis, initial symptoms may masquerade as another illness.

## Case presentation

A 20-year-old Caucasian male with no known comorbidities presented to the emergency department (ED) with a two-day history of fever, chills, body aches, dry cough, and loss of taste and smell. The patient was not vaccinated against COVID-19. He had a home COVID-19 test that turned out to be positive. The patient did not have any hypoxia and did not receive any targeted therapy for COVID-19 at the time. The patient’s oxygen levels were monitored at home and remained stable. His symptoms gradually improved and returned to the baseline 10 days later.

**Figure 1:**
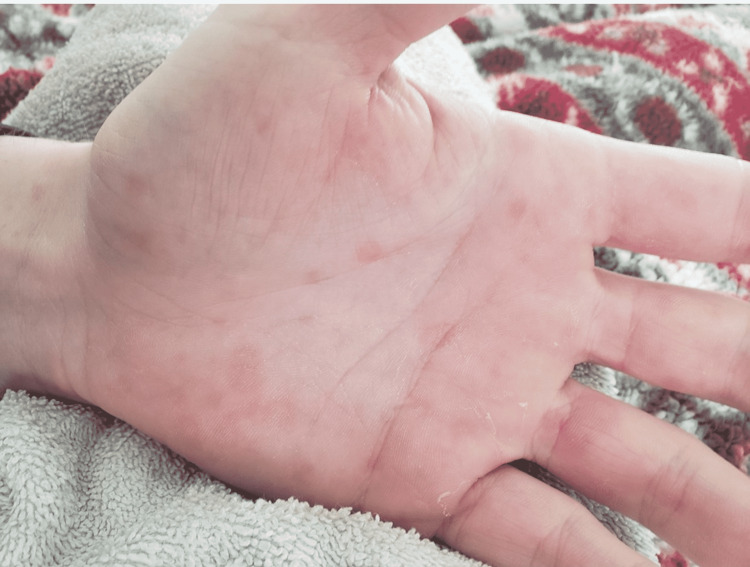
Maculopapular hand rash

Approximately four weeks after his initial symptoms, he again presented to the ED with a three-day history of fever of 102°F, body aches, dull headache, neck pain, diarrhea, and skin rash. He also complained of progressive nausea and vomiting. Upon physical examination, he was tachycardic with a heart rate of around 120 BPM, blood pressure of 105/65 mmHg, respiratory rate of 12 BPM, and oxygen saturation of 100% on room air. He was alert, oriented, and not in any distress. He had neck stiffness but no photophobia. Heart, lung, and abdominal exams were otherwise unremarkable. A generalized nontender, maculopapular, erythematous, nonblanching rash was noted involving his chest, abdomen, arms, and hands (Figure 1). He did not have any evidence of oral mucosal or conjunctivae involvement, and our initial differential diagnosis is outlined in Table 1.

**Table 1:** Differential diagnosis COVID-19: Coronavirus disease 2019.

Differential diagnosis	Supporting/excluding findings
Meningitis/Encephalitis	Headache, neck pain, and fever were concerning for meningitis or encephalitis.
Toxic shock syndrome	Fever and diarrhea are present. Risk factors are lacking.
Infectious etiology	Respiratory pathogen panel, serology, lumbar puncture, blood, and urine culture were done.
Kawasaki	Fever and rash are present. Typical features of conjunctivitis, erythema of mucous membranes, and lymphadenopathy are lacking. Patients are older than the typical age of diagnosis (5 years or younger in 90% of patients).
Macrophage activation syndrome (MAS)/Secondary hemophagocytic lymphohistiocytosis (HLH)	Fever, rash, and headache are present. Prior diagnosis of juvenile idiopathic arthritis is lacking.
Multisystem inflammatory syndrome (MIS)	Prior home COVID-19 test is positive. Fever, headache, and diarrhea are initially present.

Initial laboratory results are shown in Table 2. Blood and urine cultures were obtained. He underwent a lumbar puncture due to concerns for possible meningitis or encephalitis. He was started empirically on meropenem, vancomycin, and acyclovir. Meropenem was used due to a previous history of cephalosporin allergy. A few hours after his presentation to the ED, he decompensated and went into severe shock with tachycardia, hypotension, and hypoxia, needing aggressive fluid resuscitation, IV norepinephrine vasopressor support, and oxygen supplementation. CT chest angiography was negative for pulmonary embolism but revealed bilateral pulmonary ground-glass opacities consistent with his recent COVID-19 infection. The patient also had elevated troponins and pro-brain natriuretic peptide (pro-BNP), which were suggestive of cardiac dysfunction and possible myocarditis. Bedside point-of-care ultrasound showed decreased cardiac ejection fraction with no signs of pericardial effusion. Due to the presence of fever for more than three days, cardiac abnormalities, significantly elevated inflammatory markers (Table 1), gastrointestinal distress, and shock-like symptoms, the possibility of a multisystem inflammatory syndrome associated with COVID-19 was entertained. Intravenous steroids were initiated, and the patient was admitted to the medical intensive care unit (ICU) for further care and management where he was started on intravenous immunoglobulin (IVIG) and aspirin.

**Table 2:** Initial laboratory results

Test	Result	Reference range
Complete blood count (CBC)		
Hemoglobin (HB)	11.4	13.2-16.6 g/dL
White blood cell count (WBC)	8.0	3.4-9.6 × 10^9^/L
Platelets	149	135-317 × 10^9^/L
Coagulation studies		
International normalized ratio (INR)	1.3	0.9-1.1
Activated partial thromboplastin time (APTT)	48	25-35 s
Comprehensive metabolic panel (CMP)		
Sodium	143	135-145 mmol/L
Potassium	4	3.6-5.2 mmol/L
Chloride	106	98-107 mmol/L
Bicarbonate	29	22-29 mmol/L
Anion gap	8	7-15
Blood urea nitrogen (BUN)	16	8-24 mg/dL
Creatinine	0.88	0.74-1.35 mg/dL
Glucose	155	70-140 mg/dL
Bilirubin (Total)	1.6	≤1.2 mg/dL
Bilirubin (Direct)	1.2	≤0.0-0.3 mg/dL
Aspartate transaminase (AST)	27	8-48 U/L
Alanine transaminase (ALT)	23	7-55 U/L
Alkaline phosphatase	106	40-129 U/L
Albumin	3.1	3.5-5.0 g/dL
Calcium	7.6	8.6-10 mg/dL
Inflammatory markers		
Tumor necrosis factor (TNF)	21.2	˂10.0 pg/mL
C-reactive protein (CRP)	63	≤8.0 mg/dL
D-dimer	1923	≤500 ng/mL FEU
Ferritin	306	24-336 mcg/L
Interleukin-6 (IL-6)	46.0	˂5.0 pg/mL
Interferon-beta (IFN-β)	˂20.0	˂20.0 pg/mL
Interleukin 10 (IL-10)	10.4	˂7.0 pg/mL
Monocyte chemoattractant protein-1 (MCP-1)	127	≤198 pg/mL
Interleukin 1 beta (IL-1β)	22.9	˂20.0 pg/mL
Interferon gamma (IFN-γ)	˂60	˂60 pg/mL
Macrophage inflammatory protein-1 alpha (MIP-1α)	˂220.0	˂220.0 pg/mL
Granulocyte macrophage-colony stimulating factor (GM-CSF)	˂15.0	˂15.0 pg/mL
Interleukin-2 receptor alpha (IL-2R alpha)	2402	˂959 pg/mL
Interferon alpha (*IFN*-*α)*	˂20.0	˂20.0 pg/mL
Interleukin 18 (IL-18)	573	≤468 pg/mL
Cardiac biomarkers		
Troponin	171	≤15 ng/L
N-terminal-pro-hormone B-type natriuretic peptide (NT-proBNP)	1497	5-15 pg/mL

Transthoracic echocardiogram obtained on arrival was significant for a mobile echodensity measuring 7 mm x 50 mm in the right ventricle, concerning a thrombus. Given the possibility of a cardiac thrombus, the patient was started on a high-intensity heparin nomogram. The blood and urine cultures came back negative. The lumbar puncture results (Table 3) and an extensive microbiology workup (Table 4) were also unremarkable. He was continued on IV antibiotics with meropenem and vancomycin for concerns of an infected thrombus. The patient was subsequently evaluated by rheumatology who endorsed that he met the criteria for MIS. Continuing the steroid regimen was recommended. The rash noted on the skin improved significantly with a high dose of methylprednisolone.

**Table 3:** Lumbar puncture results HSV: Herpes simplex virus; PCR: Polymerase chain reaction.

Test	Result
Gram stain	No organisms seen
Bacterial culture, anaerobic	No growth after 7 days of incubation
Bacterial culture, aerobic + susceptibility	No growth after 5 days of incubation
HSV 1 PCR	Negative
HSV 2 PCR	Negative

**Table 4:** Microbiology results HIV-1/2: Human immunodeficiency virus 1/2; Ag: Antigen; Ab: Antibody; EIA: Enzyme immunoassay; CMV: Cytomegalovirus; DNA: Deoxyribonucleic acid; HSV-1: Herpes simplex virus 1; PCR: Polymerase chain reaction; HSV: Herpes simplex virus 2; EBV: Ebstein-Barr virus; VCA: Viral capsid antigen; IgM: Immunoglobin M; IgG: Immunoglobin G; EBNA: Ebstein-Barr virus nuclear antigen; SARS-CoV-2: Severe acute respiratory syndrome coronavirus 2; RSV: Respiratory syncytial virus.

Tests	Results
Respiratory pathogen panel	Undetected
HIV-1/-2 Ag and Ab screen	Negative
Blastomyces Ab, EIA	Negative
Histoplasma mycelial	Negative
Histoplasma yeast	Negative
Histoplasma immunodiffusion	Negative
Cryptococcus Ag screen with titer	Negative
CMV DNA detect/quant	Undetected
HSV-1 PCR	Negative
HSV-2 PCR	Negative
Syphilis total Ab with reflex	Nonreactive
EBV DNA detect/quant	Undetected
EBV VCA IgM Ab	Negative
EBV VCA IgG Ab	Positive
EBNA Ab	Positive
SARS-CoV-2 nucleocapsid total Ab	Positive
SARS-CoV-2 spike Ab	Positive
SARS-CoV-2 spike quantitative	116 U/mL
Influenza A/B	Negative
RSV	Negative

Cardiac magnetic resonance imaging (MRI) was performed to further evaluate the right ventricular density and assess for myocarditis. It did not show any evidence of myocarditis. The previously noted echodensity/mass in the right ventricle was not identified on MRI. An echocardiogram was repeated three days later, which showed an improvement in left ventricular ejection fraction (LVEF) up to 52%, and the mass seen previously was not identified. The patient showed remarkable improvement in his medical condition. Antibiotics were discontinued at this time, and a steroid taper was started. The patient was discharged home six days later with recommendations to follow up with his primary care physician.

## Discussion

SARS-CoV-2 infection is associated with MIS, a novel hyperinflammatory condition. Clinicians have faced immense challenges due to the wide variations of disease severity of SARS-CoV-2 infection. MIS-C is one of the serious complications caused by the virus. The initial cases of MIS-C were first detected in April 2020 in the United Kingdom. Several critically ill children were then noted to present with hyper-inflammation shock and COVID-19 infections as reported by the Pediatric Intensive Care Society [8]. As a result, the Royal College of Pediatricians and Child Health (RCPCH) designated it as pediatric inflammatory multisystem syndrome temporally associated with SARS-CoV-2 (PIMS-TS) [9]. Both Centers for Disease Control and Prevention (CDC) and the World Health Organization (WHO) published case definitions for MIS-C following these initial reports [10,11].

In MIS-C, the symptoms range from nausea, vomiting, abdominal pain, conjunctivitis, respiratory symptoms, neurological symptoms, rash, and lymphadenopathy [12-17]. Acute respiratory failure, shock, hypotension, myocardial dysfunction, and pericardial effusions are all common clinical findings [13,4,17-19]. The mucocutaneous symptoms of MIS-C are more common in younger children. Older children more commonly present with gastrointestinal symptoms and myocarditis [4,17]. Laboratory tests reveal elevated inflammatory markers (D-dimer, C-reactive protein [CRP], erythrocyte sedimentation rate [ESR], procalcitonin, fibrinogen, and interleukin-6), pancytopenia, elevated cardiac markers (troponin and BNP), elevated liver enzymes, hypoalbuminemia, and hypertriglyceridemia [13,4,17,20].

Case definitions for MIS-C vary between CDC and WHO [9-11] The fundamental differences are the age of the patient, the length of the fever, and whether it requires a positive SARS-CoV-2 test, or exposure. To diagnose MIS-C, the CDC requires a history of hospitalization, age < 21 years, a positive laboratory test for current or recent SARS-CoV-2 infection, or exposure to SARS-CoV-2 within the past four weeks [10]. However, MIS-C is diagnosed if the patient is less than 19 years, according to WHO [1]. There is increasing evidence that COVID-19 pathogenesis involves an array of changes including mild, acute, chronic, multi-systemic inflammatory syndromes affecting every age group [13,14,21]. Several reports of a similar syndrome in adults (MIS-A) more than 21 years of age after four weeks after initial infection with SARS-CoV-2 have been reported [11,13,14,21]. Global attention has been drawn concurrently to reports of a similar syndrome in adults without severe respiratory illness who experience cardiovascular, gastrointestinal, dermatologic, and neurological symptoms. They either had polymerase chain reaction (PCR) results that showed SARS-CoV-2 infection or had a positive antibody test showing recent infection. Positive antibodies and negative PCR results suggest that MIS-A/MIS-C are post-infectious syndromes [4]. 

At present, there is no definitive diagnosis or etiology for MIS-A. Although MIS-A patients exhibit remarkably similar clinical features to MIS-C, there may be differences in increased cardiac dysfunction severity, the incidence of thrombosis, and mortality in MIS-A [5]. Currently, there is no evidence to support a definitive treatment strategy for MIS-A, which typically requires symptomatic or supportive treatment. Glucocorticoids and intravenous immunoglobulin are commonly used to treat children with MIS-C [10,13]. It has not been shown whether these treatments have the same effectiveness in patients with MIS-A. 

Differentiating MIS-C/A from alternative diagnoses is crucial since the treatment can vary greatly. With a detailed history, physical examination, and laboratory investigation, coupled with a high level of clinical suspicion based on exposure history, clinical certainty can be achieved. MIS-C/A resembles Kawasaki disease as it is characterized by erythematous rashes, mucous membrane involvement with erythema, firm induration of limbs, conjunctivitis, and mucositis [22]. Additionally, MIS-C/A has similarities to mucocutaneous symptom complexes, such as staphylococcal toxic shock syndrome (TSS), secondary hemophagocytic lymphohistiocytosis (HLH), and macrophage activation syndrome (MAS) [13,14,17,23]. "Polymorphic" is the term used to describe the rash associated with MIS-C/A [24]. So, it is imperative to rule out other diseases characterized by fever, rash, and mucocutaneous symptoms like meningitis, encephalitis, and cellulitis. For example, they can present with some characteristics of MIS-C/A, but the infections tend to involve one organ system rather than the multisystem involvement seen in MIS-C/A. MIS-C/A can also mimic manifestations of other diseases like toxic epidermal necrolysis (TEN) and Stevens-Johnson syndrome (SJS) [25], and systemic DRESS (drug rash with eosinophilia and systemic symptoms) syndrome [26].

## Conclusions

As discussed, MIS-C/A is a diagnosis of exclusion and requires a high level of suspicion due to the numerous etiologies that manifest with similar symptoms. Our patient presented with symptoms that are highly suspicious of encephalitis or meningitis and developed cardiogenic shock within three hours of ED arrival. Clinicians should be aware of appropriate workup and that early resuscitation may help improve outcomes. There is still uncertainty concerning the chronic sequelae and long-term consequences of the disease, and further research on immunopathogenesis is imperative.
